# Production of Helvolic Acid in Metarhizium Contributes to Fungal Infection of Insects by Bacteriostatic Inhibition of the Host Cuticular Microbiomes

**DOI:** 10.1128/spectrum.02620-22

**Published:** 2022-09-01

**Authors:** Yanlei Sun, Song Hong, Haimin Chen, Ying Yin, Chengshu Wang

**Affiliations:** a Key Laboratory of Insect Developmental and Evolutionary Biology, CAS Center for Excellence in Molecular Plant Sciences, Shanghai Institute of Plant Physiology and Ecology, Chinese Academy of Sciences, Shanghai, China; b CAS Center for Excellence in Biotic Interactions, University of Chinese Academy of Sciences, Beijing, China; c School of Life Science and Technology, ShanghaiTech University, Shanghai, China; Universidade de Sao Paulo

**Keywords:** chemical biology, cuticular microbiome, entomopathogenic fungus, helvolic acid, *Metarhizium*, bacteriostatic activity

## Abstract

The nortriterpenoid helvolic acid (HA) has potent antibiotic activities and can be produced by different fungi, yet HA function remains elusive. Here, we report the chemical biology of HA production in the insect pathogen Metarhizium robertsii. After deletion of the core oxidosqualene cyclase gene in Metarhizium, insect survival rates were significantly increased compared to those of insects treated with the wild type and the gene-rescued strain during topical infections but not during injection assays to bypass insect cuticles. Further gnotobiotic infection of axenic *Drosophila* adults confirmed the HA contribution to fungal infection by inhibiting bacterial competitors in an inoculum-dependent manner. Loss of HA production substantially impaired fungal spore germination and membrane penetration abilities relative to the WT and gene-complemented strains during challenge with different Gram-positive bacteria. Quantitative microbiome analysis revealed that HA production could assist the fungus to suppress the *Drosophila* cuticular microbiomes by exerting a bacteriostatic rather than bactericidal effect. Our data unveil the chemical ecology of HA and highlight the fact that fungal pathogens have to cope with the host cuticular microbiomes prior to successful infection of hosts.

**IMPORTANCE** Emerging evidence has shown that the plant and animal surface microbiomes can defend hosts against fungal parasite infections. The strategies employed by fungal pathogens to combat the antagonistic inhibition of insect surface bacteria are still elusive. In this study, we found that the potent antibiotic helvolic acid (HA) produced by the insect pathogen Metarhizium robertsii contributes to natural fungal infection of insect hosts. Antibiotic and gnotobiotic infection assays confirmed that HA could facilitate fungal infection of insects by suppression of the host cuticular microbiomes through its bacteriostatic instead of bactericidal activities. The data from this study provide insights into the novel chemical biology of fungal secondary metabolisms.

## INTRODUCTION

Entomopathogenic fungi such as the ascomycete Metarhizium and *Beauveria* species can produce a plethora of secondary metabolites (SMs) with different activities (1). Chemical biology investigations have revealed that the SMs produced by Metarhizium and *Beauveria* species, such as the cyclodepsipeptides destruxins, beauverolides, and beauvericin and the polyketides oosporein and tenellin, contribute to fungal virulence against insect hosts by invasion or evasion of host immunities (2–6). The pigment oosporein produced by *Beauveria* has antibiotic activity and has also been shown to inhibit bacterial proliferation in insects during and after fungal killing of insects (7, 8). The alkaloid mycotoxin swainsonine produced by plant endophytes and Metarhizium species is implicated in mediating defenses against plant-grazing and insect-feeding animals (9, 10). Fungal SMs can thus mediate chemical biology and ecology beyond the interactions with hosts.

The nortriterpenoid helvolic acid (HA) was first identified from the mammalian pathogenic fungus Aspergillus fumigatus (11) and has been characterized with potent antibiotic activities against different Gram-positive (G+) bacteria (12). It was later found that HA can also be produced by different ascomycete endophytic fungi and plant pathogens, such as Fusarium, *Penicillium*, and *Sarocladium* species (13–15). Metarhizium species can also form endophytic and or rhizosphere relationships with plants (16), and the production of HA has been reported in Metarhizium anisopliae (17). It has been shown in A. fumigatus that HA is biosynthesized by the oxidosqualene cyclase (OSC, i.e., HelA) biosynthetic gene cluster (BGC) through the cyclization of 2,3-oxidosqualene by OSC plus the functions of the tailoring enzymes cytochrome P450s, acyltransferases (ATs) and 3-ketosteroid-Δ^1^-dehydrogenase (KSTD) (18, 19). The chemical ecology of HA production remains unclear.

In contrast to *per os* infections of insects by pathogenic bacteria and viruses, fungal pathogens infect insects through spore germination on host cuticles and the penetration of exoskeletons (20, 21). Similar to the protective barrier functions of the human skin and plant phyllosphere microbiotas (22–24), diverse insects, such as ants, wasps, and beetles, have evolved the ability to assemble defensive ectosymbiotic bacteria on body surfaces to defend against fungal parasitic infections (25–28). We recently showed that the surface microbiotas assembled on Drosophila melanogaster are beneficial to hosts by inhibiting the spore germinations of entomopathogenic fungi on fly surfaces (29). This prompted the question of which strategies are employed by insect pathogens to pave the way for cuticular penetrations and infections.

In this study, we report that HA biosynthesis in Metarhizium species contributes to fungal topical infection of insects. HA can effectively inhibit the proliferation of the G+ bacteria isolated from D. melanogaster and facilitate fungal spore germinations and penetration of cellophane membranes in the presence of difference G+ bacteria. Quantitative microbiome analysis after inoculation of fungal strains indicated that HA production in Metarhizium robertsii can inhibit the insect cuticular microbiomes to facilitate fungal infections by exerting bacteriostatic rather than bactericidal activities.

## RESULTS

### HA production by different Metarhizium species.

**FIG 1:**
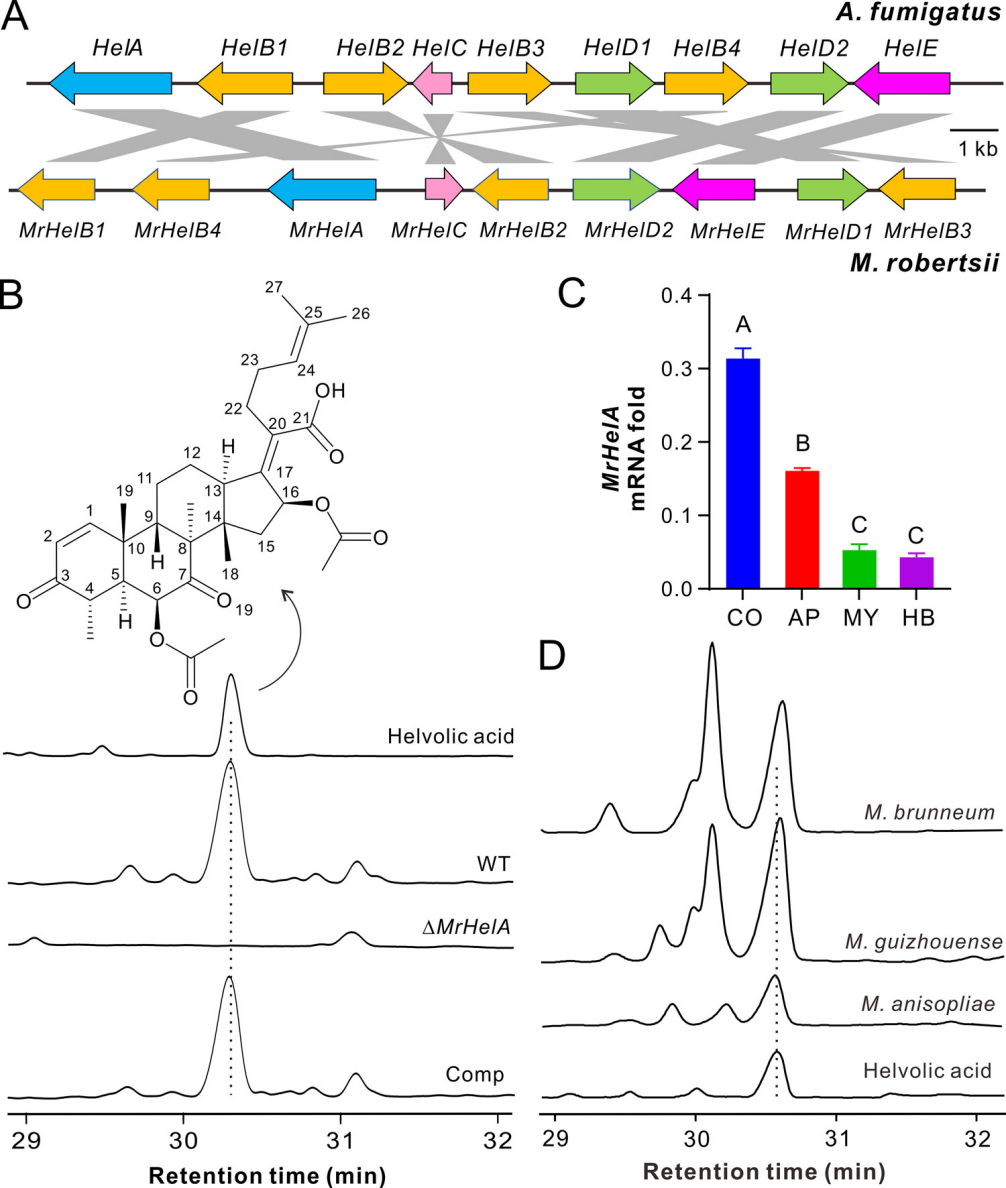
Conservative production of helvolic acid (HA) by different Metarhizium species. (A) Mesosyntenic relationship between the HA BGCs in A. fumigatus and *M. robertsii*. Gene models in the same color represent coding of proteins in a family. The putative function of each gene is listed in Table S1. (B) Verification of HA production by *M. robertsii*. Comp, complemented strain of the Δ*MrHelA* mutant. The WT and mutant strains were used to infect last-instar larvae of the wax moth, and the mycosed insect cadavers were extracted with ethyl acetate for HPLC analysis. (C) Differential expression of *MrHelA* by *M. robertsii*. CO, conidia harvested from the 2-week-old PDA plates; MY, mycelia harvested from the 3-day-old SDB culture; AP, appressorium cells induced on soldier fly wings for 18 h; HB, hyphal body cells harvested from the hemolymph of *Galleria* larvae after injection for 3 days. Values are means and standard deviations (SD). The difference between samples was determined by one-way ANOVA. Different letters above columns indicate differences with a *P* value of <0.01. (D) Verification of HA production by different Metarhizium species. The fungi were inoculated on rice medium for 2 weeks for metabolite extractions.

Our genome survey indicated that the conserved OSC BGC is present in *M. robertsii* and shows a mesosyntenic relationship with that of A. fumigatus (Fig. 1A). For example, the putative OSC MrHelA is highly conserved to HelA (59% identity at the amino acid level), and each contains a squalene/oxidosqualene cyclase domain. This gene cluster is also present in the genomes of different Metarhizium species (see Table S1 in the supplemental material), each of which has a broad host range (30). A BLASTP search indicated that the conserved OSC enzymes are also encoded by the other Aspergillus fungi, plant endophytes, and pathogens of the phylum Ascomycota as well as the basal fungal species belonging to the phyla Zoopagomycota and Mucoromycota (Fig. S1). However, intriguingly, the homologues of tailoring-enzyme genes such as the cytochrome P450 genes *HelB1*/*MrHelB1* and KSTD genes *HelE*/*MrHelE* either are absent or have nonclustered homologues in some OSC-containing ascomycete fungi and the basal fungal species (Fig. S1B and C), suggesting that these fungi may not produce HA.

To determine HA production in Metarhizium, we performed deletion and gene complementation of *MrHelA* in *M. robertsii*. The mutants obtained had no obvious growth defects compared with the wild-type (WT) strain (Fig. S2A). There was also no obvious difference in resistance against salt stresses between the WT and mutant strains (Fig. S2B). After infection of insects and extraction of mycosed (fungus-mummified insect cadavers) wax moth (Galleria mellonella) cadavers using ethyl acetate, high-performance liquid chromatography (HPLC) analysis revealed that a compound peak was absent in the Δ*MrHelA* sample in contrast to those of the WT and the complemented mutant (Comp) (Fig. 1B). This compound was purified from the WT sample and structurally identified as HA (Table S2), confirming that *MrHelA* is responsible for HA production in *M. robertsii*. Gene expression analysis revealed that *MrHelA* was most highly transcribed by *M. robertsii* in conidia, which was followed by its expression in appressorium cells induced on fly wings, mycelial cells harvested from the saprophytic broth, and hyphal body cells harvested from the caterpillar body cavity (Fig. 1C). Consistent with the presence of the conserved BGC, we verified that HA could also be produced by the species Metarhizium brunneum, Metarhizium guizhouense, and *M. anisopliae* (Fig. 1D).

### Production of HA facilitates natural fungal infection of insects.

**FIG 2:**
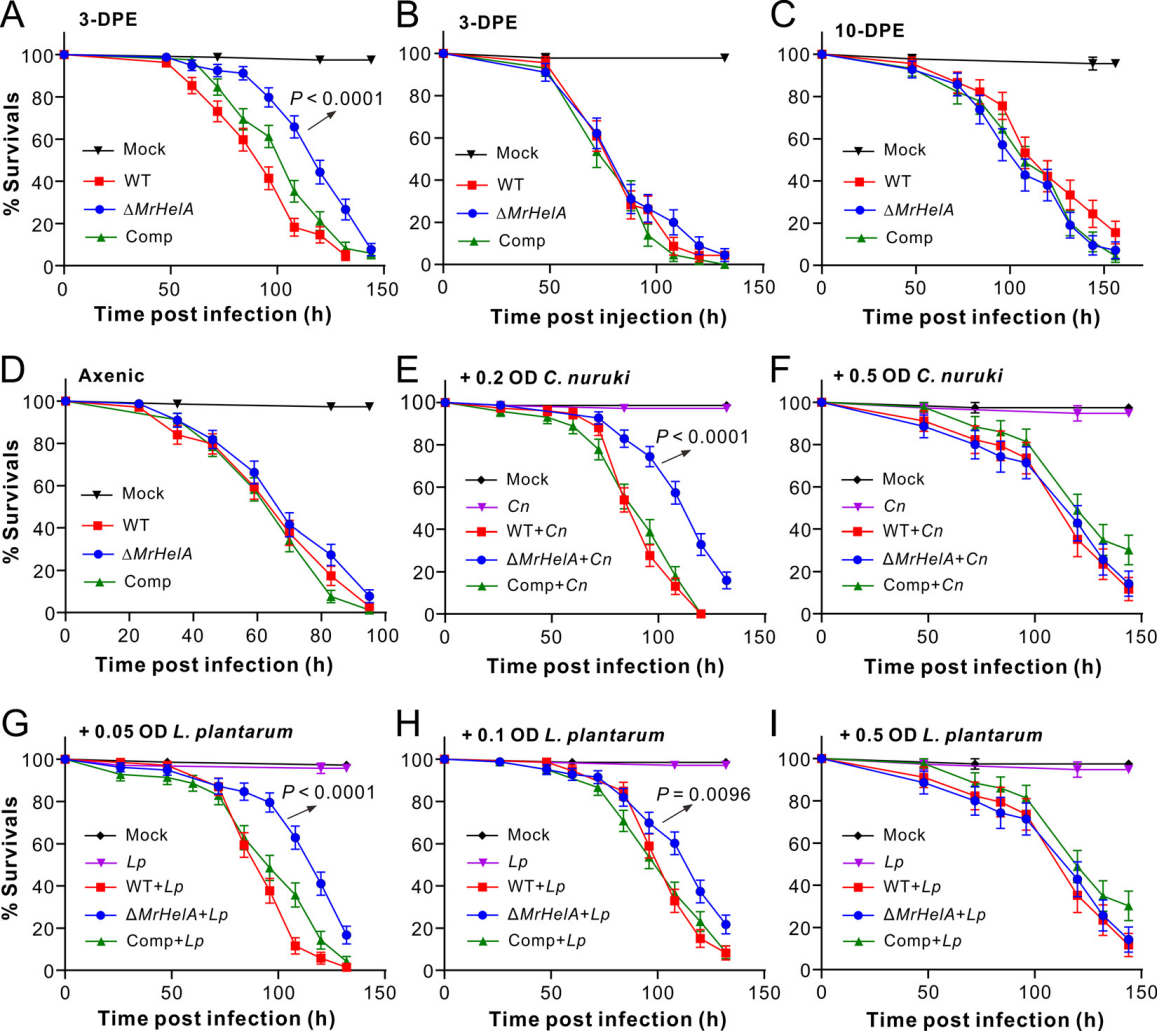
Survival of *Drosophila* males after different treatments. (A and B) Survival of 3-DPE male flies after topical infections (A) and injections (B) with different Metarhizium strains. (C) Survival of 10-DPE male flies after topical infections. (D) Survival of 3-DPE axenic male flies after topical infections. (E and F) Survival of 3-DPE axenic male flies after topical infections with different strains plus the addition of *C. nuruki* (*Cn*) cells in spore suspensions at an OD_600_ of 0.2 (E) or 0.5 (F). (G to I) Survival of 3-DPE axenic male flies after topical infections with different strains plus the addition of *L. plantarum* (*Lp*) cells in spore suspensions at an OD_600_ of 0.05 (G), 0.1 (H), or 0.5 (I). More than 70 flies were used for each treatment. Values are means and standard errors of the means (SEM). The difference in survival between the treatments was determined by log-rank tests. The *P* values with arrows indicate the difference between the WT and Δ*MrHelA* treatments. Solutions in 0.05% Tween 20 with and without the same number of bacterial cells were included as controls.

We next performed both topical infection and injection assays of different insects with the WT and mutant strains of *M. robertsii*. It was found that, relative to the WT strain, the deletion of *MrHelA* could significantly (log rank test: χ^2^ = 35.31, *P* < 0.001) impair fungal topical infection of D. melanogaster male adults that were used 3 days posteclosion (DPE) (Fig. 2A). An approximately 25% increase of the median lethal time (LT_50_) was found for the flies treated with the Δ*MrHelA* strain in relation to the WT strain. In contrast, fly survival differences were not observed after injection of 3-DPE males with different fungal strains (Fig. 2B). Similar patterns were obtained during the topical infection and injection assays of spotted wing drosophila (Drosophila suzukii) and wax moth larvae (Fig. S3). For example, a substantial difference was evident between the WT and Δ*MrHelA* strains during topical infection (χ^2^ = 4.93, *P = *0.026) but not injection of the 3-DPE *D. suzukii* males. In addition to the survival difference (χ^2^ = 6.47, *P = *0.011) between caterpillars treated with the WT and the null mutant, a higher number of the wax moth cadavers killed by the Δ*MrHelA* strain was corroded by bacteria without mycosis than those killed by the WT (Fig. S4A and B). Quantification of HA production showed that the caterpillar cadavers mycosed by WT (290.0 ± 51.4 μg/g) and Comp (266.3 ± 44.27 μg/g) strains accumulated relatively high amounts of HA but not those killed by the Δ*MrHelA* strain (Fig. S4C). Intriguingly, however, we also found that the topical infection of 10-DPE flies abrogated the difference in the survival of D. melanogaster after challenge with different strains (Fig. 2C).

We then generated axenic flies for gnotobiotic assays using fungal spore suspensions with and without the addition of cells of Lactiplantibacillus plantarum and Corynebacterium nuruki that were isolated from *Drosophila* surfaces as dominant G+ bacterial species (29). In contrast to the differential survival rates of the conventionally reared 3-DPE flies, no statistical difference in survival was observed for the 3-DPE axenic flies after being topically treated with the WT and mutant spores (Fig. 2D). After preliminary trials, the spore suspensions were added with *C. nuruki* cells at final optical densities at 600 nm (OD_600_) of 0.2 and 0.5 for topically treating the germfree male flies. The results indicated that the added cells at an OD_600_ of 0.2 (χ^2^ = 35.31, *P* < 0.001) but not 0.5 (χ^2^ = 0.041, *P = *0.84) resulted in a significant difference in fly survival between the WT and Δ*MrHelA* treatments (Fig. 2E and F). Likewise, the additions of *L. plantarum* cells at OD_600_ of 0.05 and 0.1 but not 0.5 in spore suspensions resulted in a significant difference in fly survival rates between the WT and Δ*MrHelA* strains (Fig. 2G to I). Thus, the addition of small amounts of bacterial cells could substantially increase the survival of flies treated with the Δ*MrHelA* strain relative to those treated with the WT and Comp strains. In contrast, the addition of large amounts of bacterial cells could level the difference between strains. The results indicated that HA contribution to Metarhizium infection of insects would be associated with the inoculum of host surface bacteria.

### HA production facilitates fungal spore germination by outcompeting different bacteria.

**FIG 3:**
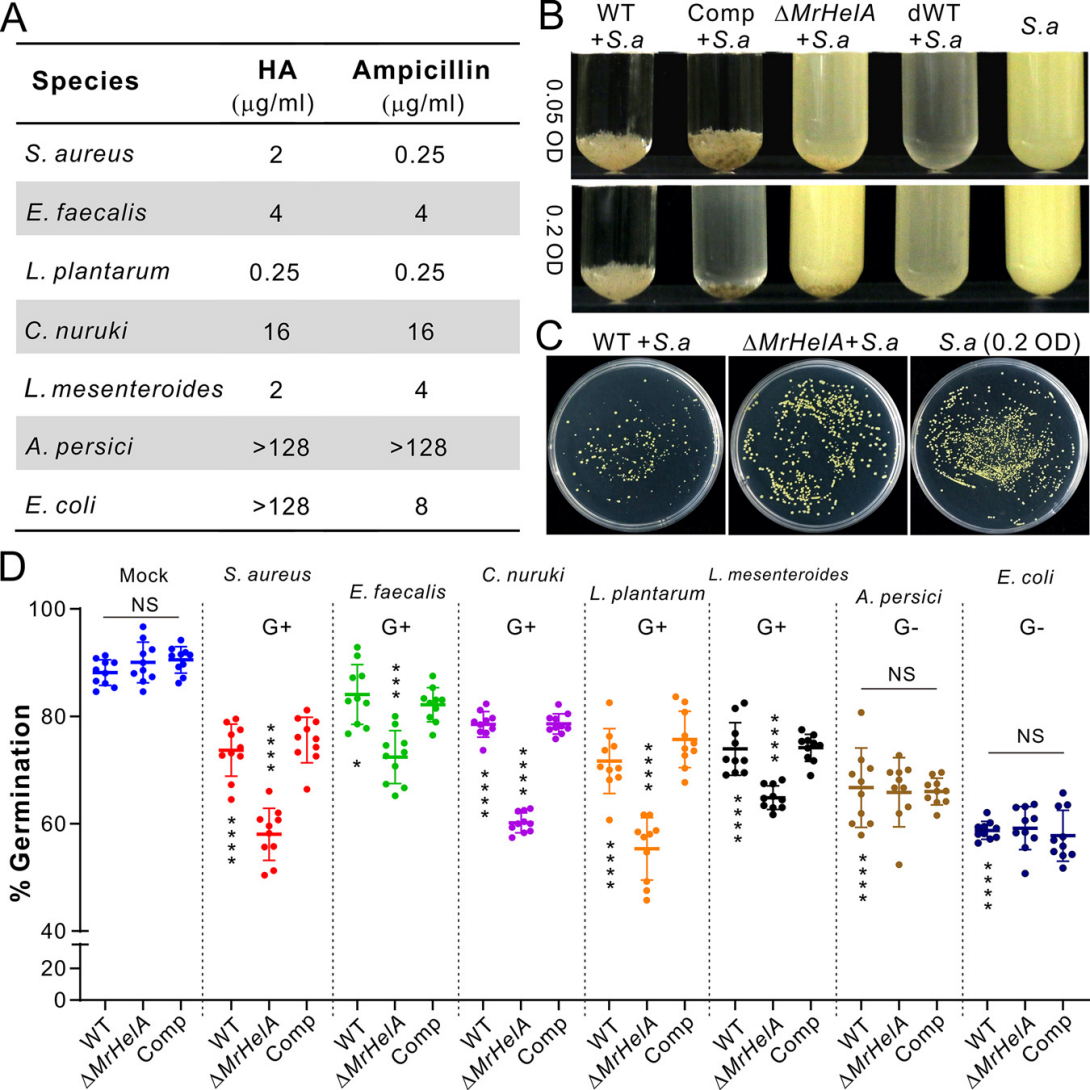
MIC test of HA against different bacteria and cross-inhibition assays between fungi and bacteria. (A) MICs of HA against different bacteria. Ampicillin was used as a control. (B) Differential inhibition of S. aureus (*S. a*) growth by the WT and mutant strains in cocultures. The spores of different Metarhizium strains were cocultured with S. aureus cells at different initial amounts in LB broth for 24 h. dWT, dead spores of WT strain. (C) Features of S. aureus colony formation after different treatments. The S. aureus cells (all at a final OD_600_ of 0.2) were mixed with or without the WT and Δ*MrHelA* spores for inoculation on LB agar for 24 h. (D) Differential inhibition of the WT and mutant spore germinations by different bacteria. The spores of Metarhizium strains were cocultured with different bacteria for 12 h to determine the spore germination rate of each strain. Values are means and SD. The differences between samples were determined by two-tailed Student's *t* test. The asterisks above the values for the Δ*MrHelA* strain show the within-group difference between the WT/Comp and the Δ*MrHelA* strains. The asterisks below the WT values show the difference between the mock-treated control and individual bacterial treatment of the WT. *, *P* < 0.05; ***, *P* < 0.001; ****, *P* < 0.0001; NS, not significant.

We next examined the MIC of HA against different bacteria, including the dominant G+ and G− species isolated from *Drosophila* body surfaces (29). As a result, we found that HA’s effects were equivalent to or better than those of ampicillin against the G+ bacteria *L. plantarum*, *C. nuruki*, Enterococcus faecalis, and Leuconostoc mesenteroides isolated from flies as well as against the common experimental G+ bacterium Staphylococcus aureus. In contrast, HA was largely ineffective toward the G− bacteria Acetobacter persici and Escherichia coli (Fig. 3A). We also performed cross-inhibition assays by coculturing fungal spores with the cells of S. aureus in Luria-Bertani (LB) broth. Both the WT and Comp strains but not the Δ*MrHelA* strain could inhibit growth of S. aureus inoculated with bacterial cells at OD_600_ values of 0.05 or 0.2 (Fig. 3B). Consistent with the high expression of *MrHelA* in fungal conidia shown above, we found that the heat-killed WT spores could effectively inhibit bacterial growth, confirming fungal production and storage of HA in conidial spores. The expression of *MrHelA* could also be quickly upregulated in *M. robertsii* by coculturing with S. aureus (Fig. S4D). The coinoculations on solid LB agar similarly revealed that the WT spores could apparently inhibit the colony formation of S. aureus compared with the Δ*MrHelA* and mock-treated controls (Fig. 3C).

We further examined fungal spore germinations in the presence or absence of bacterial cells (Fig. S5). Not surprisingly, the addition of either the G+ or G− bacterial cells could substantially inhibit the germination of the WT and mutant spores compared with their performances in the pure LB medium (Fig. 3D). Among fungal strains, the difference in germination rates was not apparent for fungi inoculated in the blank LB medium and LB plus the G− bacteria *A. persici* and E. coli (Fig. 3D). Otherwise, the germination of Δ*MrHelA* spores was significantly (two-tailed Student's *t* test, *P* < 0.001) delayed relative to the WT and Comp strains in the presence of all the G+ bacteria used in the experiments described above (Fig. 3D). The data demonstrated that both the G+ and G− bacteria, especially the latter, could deter fungal spore germination, while HA production enabled the fungus to battle different G+ bacteria. Consistent with this, and also in support of infection assay results, the addition of G+ bacterial cells could impair the membrane penetration ability of Δ*MrHelA* but not WT and Comp strains compared with the mock-treated control. However, the addition of cells of the G− bacteria *A. persici* and E. coli could render both the WT and mutants unable to penetrate cellophane membranes (Fig. S6).

### Differential manipulation of *Drosophila* surface bacterial loads by fungal strains.

**FIG 4:**
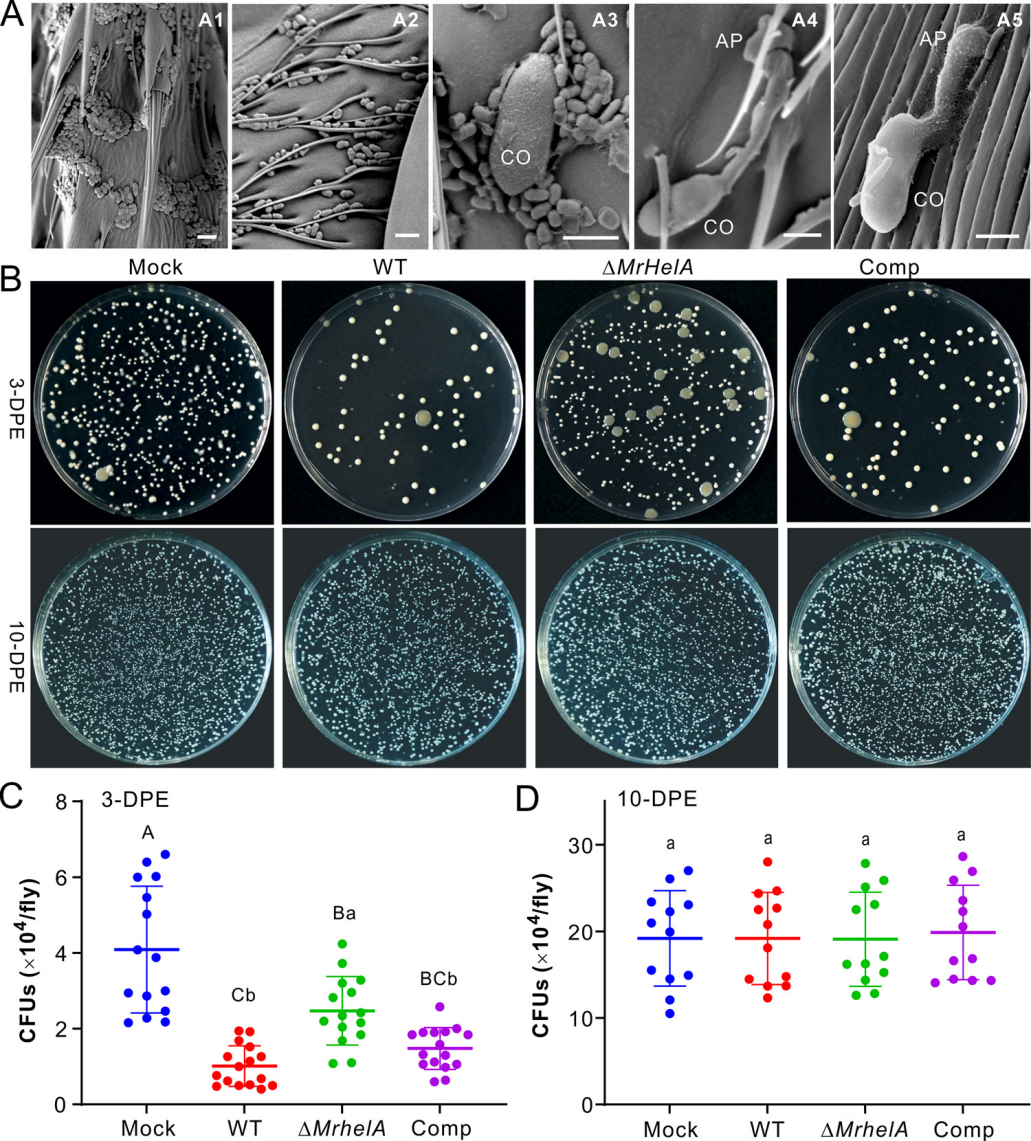
Manipulation of *Drosophila* cuticular bacterial loads by different fungal strains. (A) SEM observation of *Drosophila* surfaces. Dense loads of bacterial cells were observed on the tarsal segments (A1) and abdomen (A2) of 10-DPE flies. Once in contact with multiple bacterial spores, germination of the conidium (CO) was inhibited (A3). Otherwise, spores could germinate to form appressoria (AP) with no bacterial contact (A4) or in contact with a few bacterial cells (A5). Bar, 5 μm. (B) CFU formation of the bacteria washed from the body surfaces of 3-DPE (top) and 10-DPE (bottom) male flies after treatment with different fungal strains. The wash solutions (10 flies in 1 mL of PBS buffer) were diluted 10 times prior to being plated on the LB agars. (C and D) Determination of the fly surface bacterial CFU after topical infection of 3-DPE (C) and 10-DPE (D) male flies. After treatment for 16 h, the flies were anesthetized and washed for plating and CFU counting. Ten male flies were collected and washed as an independent replicate. Values are means and SD. One-way ANOVA was conducted to determine the difference between treatments. Different letters above values show the difference with *P* values of <0.01 (capital) and <0.05 (lowercase).

We next performed scanning electron microscopy (SEM) analysis of the 10-DPE fly surfaces with and without application of fungal spores. Consistent with our previous findings (29), large amounts of bacterial cells were found on the tarsal and body surfaces of flies. Once in contact with multiple bacterial cells, germination of the *M. robertsii* spore was inhibited. Otherwise, the spores (even in contact with a few bacterial cells) could germinate to produce the infection structures appressoria (Fig. 4A). After the topical treatment of 3-DPE male flies for 18 h, washing fly body surfaces followed by plating for bacterial colony formation demonstrated the clearer inhibition of bacterial proliferation by the WT and Comp strains than by the Δ*MrHelA* strain. However, no obvious difference between treating the 10-DPE flies with the control and fungal inoculations was seen (Fig. 4B). The comparison of CFU revealed that, relative to the mock-treated control, the inoculation of either fungal strain could substantially (one-way analysis of variance [ANOVA], *P* < 0.05) reduce the number of CFU on 3-DPE flies (Fig. 4C). In comparison, substantially fewer bacterial CFU were formed after treatment of the flies with the WT and Comp strains than with the Δ*MrHelA* strain (Fig. 4C). In contrast, no statistical difference in CFU numbers was observed after the inoculation of 10-DPE flies with the WT and mutant strains in reference to the mock-treated control (Fig. 4D).

### Quantitative microbiome analysis shows the bacteriostatic effect of HA on fly cuticular microbiotas.

**FIG 5:**
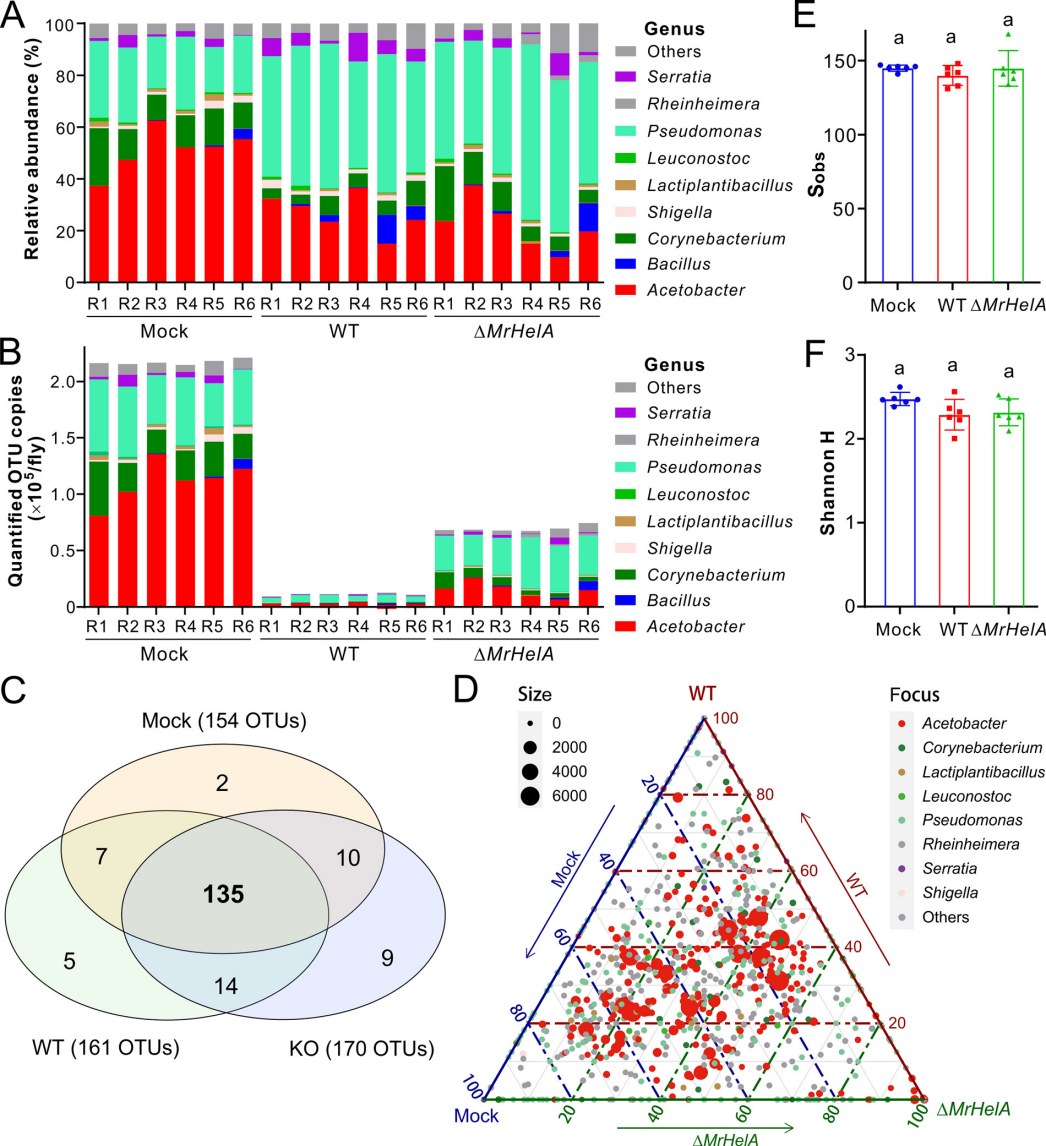
Suppression of the *Drosophila* cuticular microbiomes by *M. robertsii*. (A and B) Quantitative analysis of the fly surface microbiomes showing the relative (A) and quantified (B) abundance variations between different treatments at the bacterial genus level. 3-DPE male flies were immersed in 0.05% Tween 20 (Mock) and spore suspensions of the WT and Δ*MrHelA* strains for 30 s. After treatment for 16 h, the flies were collected (10 per replicate) for washing off surface bacteria for 16S rRNA amplification and library sequencing. Quantification analysis was conducted based on the addition of the synthetic spike-in standard. (C) Venn diagram analysis showing the detected bacterial OTUs largely shared between treatments. (D) Ternary plot analysis showing the core bacterial taxa shared between samples. (E and F) Nonvariation of the total observed OTU numbers (S_obs_) (E) and Shannon H indices (F) between treatments. One-way ANOVA was conducted, and the same letter above each column represents nonsignificant variation between samples.

We further performed quantitative microbiome analysis of 3-DPE flies after treatments with the WT and Δ*MrHelA* strains to determine the difference of the bacterial operational taxonomic units (OTUs). No obvious difference in relative abundance was observed at the phylum level between the fungal inoculations and mock-treated control (Fig. S7A). At the genus level, however, numbers of *Acetobacter* bacteria decreased while those of Pseudomonas bacteria increased after the inoculation of either WT or Δ*MrHelA* spores compared with the control (Fig. 5A). After normalization to the spike-in standard, the bacterial loads were found to have an approximately 20-fold reduction on flies after treatment with the WT spores and around a 3-fold reduction on the flies challenged with the Δ*MrHelA* spores at both the bacterial phylum and genus levels relative to mock-treated controls (Fig. 5B; Fig. S7B). Intriguingly, our Venn diagram analysis revealed that the detected OTUs were largely shared (>80%) among the control, WT, and Δ*MrHelA* treatments (Fig. 5C). A ternary plot analysis confirmed that the core bacterial taxa were shared between samples (Fig. 5D). We also calculated the α-diversity indices and found that there were no statistical differences between the control and fungal inoculations in terms of the total detected OTUs and Shannon H index (Fig. 5E and F). Taken together, the results suggested that HA might function as a bacteriostatic rather than bactericidal antibiotic. To confirm this, we performed bacterial survival assays by challenging bacteria with 1×, 2×, and 4× MIC of HA for 20 h. Reinoculation of each sample on the LB plates indicated that, similar to the mock-treated control, the examined bacteria, *L. plantarum* and *A. persici*, were still alive to form colonies, while a bactericidal effect was observed for the non-fly-origin bacterium S. aureus after treatment with >2× MIC of HA (Fig. S8). The minimum bactericidal concentration (MBC) of HA was more than 4-fold its MIC against the fly-origin bacteria. The data thus support the bacteriostatic effect of HA against these bacteria based on the standard of an MBC/MIC ratio of >4 (31).

## DISCUSSION

In this study, we found that Metarhizium species with broad host ranges carry the conserved OSC BGC to produce HA. The deletion of *MrHelA* in *M. robertsii* followed by insect bioassays revealed that HA production contributes to natural fungal infections of different insects. The combined data revealed that HA production enables *M. robertsii* to carry out bacteriostatic rather than bactericidal inhibition of insect cuticular bacteria to facilitate the fungal topical infection process.

We confirmed that the conserved HA BGC is present in the genome of generalist Metarhizium species and these fungi could produce HA. Intriguingly, however, this gene cluster is absent in specialist Metarhizium fungi with narrow host ranges (30). A similar pattern is observed in Aspergillus species; unlike A. fumigatus, most aspergilli, such as Aspergillus nidulans and Aspergillus oryzae, lack this BGC. Horizontal gene transfer (HGT) has been suggested in association with the patchy distribution of HA BGC in different fungi (12). Without the ability to produce the antibiotic HA, the specialist Metarhizium species might have evolved the alternative strategy to combat the host cuticular microbiomes; this requires future investigation.

It has been found that A. fumigatus can produce HA and more than 20 derivatives of HA, and three of them, such as 6-deacetyl-helvolic acid, have even more potent antibacterial activities than HA against S. aureus (19). Given the high degree of conservation between the *HelA* and *MrHelA* BGCs, it is possible that these derivatives can be similarly produced by Metarhizium species to jointly mediate the antimicrobiome effects, but this remains to be determined. The findings that *MrHelA* is highly transcribed in Metarhizium spores and quickly inducible in the presence of bacterial cells would benefit the spores by enabling them to initiate life cycles in harsh niches. The production of HA by a few Aspergillus strains and endophytes may similarly be involved in manipulating bacterial abundance in microniches.

Fungal entry into the insect body cavity requires proteases to degrade the protein- and chitin-rich cuticles (20, 21). In addition, our combinational insect bioassay and quantitative microbiome analyses provided strong evidence that HA production in *M. robertsii* contributes to fungal infection of insects by outcompeting host cuticular bacteria to facilitate fungal spore germination, appressorium differentiation, and cell entry into hosts. Likewise, the triterpenes produced by *Arabidopsis* contribute to regulating the hemostasis of root microbiotas (32, 33). It has also been found that terpene-rich lavender oil could inhibit G+ bacteria more efficiently than G− bacteria of facial skin microbiotas (34). The plant-pathogenic fungus Verticillium dahliae employs antimicrobial peptides (AMPs) to facilitate fungal colonization of plant roots by manipulating microbiomes in soils (35). Our quantitative microbiome analysis indicated that, relative to the mock-treated control, the inoculation of Δ*MrHelA* spores also led to a substantial reduction of the fly cuticular bacterial loads, which would suggest that additional factors other than HA might be involved in inhibiting insect surface microbiotas; this requires further investigation. For example, it has been reported that the defensin-like AMPs are encoded by Metarhizium fungi (36). In addition, there are more than 50 SM clusters present in the genome of *M. robertsii* (37), and additional antibiotics are likely produced by this fungus. For example, the polyketide pseurotin A can be produced by *M. robertsii* (38), and the compound has been demonstrated to have antibiotic activities against both G+ and G− bacteria (39).

The defensive ectomicrobiome of leaf-cutting ants, especially the key bacterial members *Pseudonocardia* and *Streptomyces*, can produce antifungal compounds to inhibit fungal spore germination (25, 26, 28). Strains of *L. plantarum* have been developed as potential probiotics in the food industry (40). Future investigations are still required to determine the antifungal components produced by *Drosophila* cuticular microbiotas.

Antibiotics are either bactericidal or bacteriostatic based on their abilities to kill bacteria or inhibit bacterial growth, with an MBC/MIC ratio of <4 being bactericidal and a ratio of >4 being bacteriostatic (31, 41). Consistent with previous findings (12), our data confirmed the bacteriostatic effect of HA against the bacteria isolated from *Drosophila*. This kind of effect may benefit the maintenance of the bacterial diversity after fungal killing and mycosis of insects. Regarding the machinery of bacteriostatic antibiotics, studies have shown that the antistaphylococcal agent fusidic acid, a structure analog of HA, can bind to the bacterial elongation factor to inhibit protein biosynthesis (42). The compounds targeting bacterial methionyl-tRNA synthetase to block protein synthesis are also bacteriostatic (31). The exact mechanism of the HA bacteriostatic effect remains to be determined.

Even though HA has potent bacteriostatic activities against the G+ bacterial species isolated from D. melanogaster, the topical infections of 10-DPE flies and gnotobiotic assays with the addition of large amounts of bacterial cells could all abrogate the difference in fly survival between the WT and Δ*MrHelA* strains. Thus, similar to the inoculum effect of different antibiotics (43), the presence of a large number of bacterial cells could limit the competing ability of fungal spores with the production of a specific amount of HA. The G− bacteria against which HA is ineffective, such as *Acetobacter* and *Gilliamella*, are present in greater numbers on the cuticles of older flies (29, 44), which might additionally result in the lack of difference in survival of 10-DPE flies after infections by the WT and null mutants of *M. robertsii*. Our data reinforce the importance of applying mycoinsecticides for control of insect pests at their early developmental stages (29).

In conclusion, we report that the production of HA in Metarhizium species can facilitate fungal infection of insects by bacteriostatic inhibition of the host cuticular microbiotas. The results of this study paint an interesting picture of the intimate interactions between the fungal parasites and insects with the amalgamation of bacterial competitors that have been overlooked before.

## MATERIALS AND METHODS

### Microbial strains and maintenance.

Various Metarhizium species were used in this study, including the WT strains *M. robertsii* ARSEF 2575, *M. guizhouense* ARSEF 977, and *M. anisopliae* ARSEF 549. Fungal strains were maintained on potato dextrose agar (PDA; BD Difco) at 25°C for 2 weeks. The WT and mutant strains of *M. robertsii* were also inoculated on PDA with and without the addition of different salts for stress challenges. For RNA extractions, the WT strain of *M. robertsii* was grown in Sabouraud dextrose broth (SDB; BD Difco) for 3 days in a rotary shaker at 200 rpm and 25°C. The spores of *M. robertsii* were also induced on black soldier fly (Hermetia illucens) wings for appressorium induction and RNA extraction (45). Different Metarhizium species were also inoculated on rice medium for 2 weeks for sporulation and HA extraction. Bacterial species previously isolated from the body surface of D. melanogaster were used for cross inhibition assays, including the G+ bacteria *L. plantarum*, *C. nuruki*, E. faecalis, and *Leuconostoc mesenteroides* and the G− bacterium *A. persici* (29). The commonly used experimental bacteria S. aureus (G+) and E. coli (G−) were also used. Bacteria were cultured in Luria-Bertani (LB) broth at 37°C or de Man, Rogosa and Sharpe (MRS) broth (Oxoid) at 30°C.

### *MrHelA* expression assays.

RNA samples of *M. robertsii* were extracted using the RNeasy plant minikit (Qiagen, Germantown, MD) from spores harvested from 2-week-old PDA cultures, mycelia harvested from SDB, and appressorium cells induced on soldier fly wings. Wax moth larvae were individually injected with a spore suspension (10 μL containing 1 × 10^7^ conidia/mL in 0.05% Tween 20) for 3 days for collection of insect hemolymph samples on ice. Fungal cells (hyphal bodies) were then separated for RNA extraction by centrifugation in Centricoll (Sigma-Aldrich) at 4°C (46). To examine the induction of *MrHelA* expression by bacteria, we germinated the WT strain spores in LB broth for 24 h, and the samples were divided into aliquots of 20 mL each. Fresh cells of S. aureus were then added at a final OD_600_ of 0.4 in each sample for incubation in a rotary shaker for different times at 25°C and 210 rpm prior to collection for extracting fungal RNAs. First-strand cDNA of each sample was converted using 1 μg of total RNA with the RT master mix kit (TransGen Biotech, China), and quantitative PCR analysis of *MrHelA* expression was conducted using a SYBR mix (Toyobo, Japan). The β-tubulin gene of *M. robertsii* was amplified and used as a reference (47).

### Gene clustering and phylogeny analysis.

Based on the previously obtained genome information for Metarhizium species (30), the BGCs for secondary metabolism were predicted for each species using the program antiSMASH v. 6.0 (48). The putative HA cluster was determined based on the similarity and comparative structure analysis with the HA BGC reported in A. fumigatus (19, 49). The HelA, HelB1, and HelE orthologs from other fungal species were retrieved by a BLASTP search, and the protein sequences were aligned using Clustal X (50). Neighbor-joining trees were generated using the software MEGA v. 11 with 500 bootstrap replicates and the Dayhoff model of amino acid substitution (51).

### Gene deletion and complementation.

To determine the biosynthesis of HA by the homologous *MrHelA* BGC in *M. robertsii*, we deleted *MrHelA* by homologous replacement as described before (52). In brief, the 5′ and 3′ flanking regions of *MrHelA* were amplified using the respective primer pairs (Table S3), and the purified products were cloned into the binary plasmid pDHt-Bar (with a *Bar* gene conferring resistance against glufosinate ammonium). The vector was used for the *Agrobacterium-*mediated transformation of the *M. robertsii* WT strain. The drug-resistant colonies were verified by PCR using two types of primer pairs targeting the flanking region and deletion region. For gene rescue, the full open reading frame of *MrHelA* was amplified together with its promoter region (ca. 1.5 kb upstream of the start codon), and the purified product was cloned into the binary vector of pDHt-Sur (with a *Sur* gene conferring resistance against sulfonylurea) (45). The obtained plasmid was then used to transform the null mutant of *MrHelA*.

### HA extraction and chromatography analysis.

Different Metarhizium species were grown on the rice medium for 2 weeks, and the cultures were dried at 60°C for overnight and extracted with ethyl acetate. In particular, a large amount (2 kg) of cultures was prepared for the WT strain of *M. robertsii* for compound purification and structure identification. To detect HA production in insects, we individually injected last-instar larvae of wax moths with the spore suspensions (20 μL per insect containing 3 × 10^5^ conidia/mL) of the WT and mutants of *M. robertsii*. After insect death, the cadavers were kept in a moisturized condition (relative humidity > 85%) for mycosis for 10 days. The mycosed cadavers were then freeze-dried and homogenized. Samples (3 g each) were defatted in sterile water (300 mL) and then extracted with ethyl acetate. The extracted samples were dried by vacuum evaporation and redissolved in acetonitrile. For compound purification, the sample (150-μL aliquots) was loaded into a preparative HPLC system (LC-20 AD; Shimadzu, Japan) equipped with a C_18_ column (particle size, 5 μm; 10 by 250 mm; Athena, China). Analytic HPLC analysis was performed using a small C_18_ column (particle size, 5 μm; 4.6 by 250 mm). The eluates were maintained at a flow rate of 3 mL/min for purification and 1 mL/min for analytic analysis with deionized water and acetonitrile (15 to 100%), and monitored using a diode array detector at 190 nm (5). The HA standard (Cayman Chemical, Ann Arbor, MI) was dissolved in dimethyl sulfoxide as a stock solution and included in a parallel analysis. The standard was also used for generation of standard curves for estimating the HA content produced in insect cadavers. The nuclear magnetic resonance (NMR) spectrum data of the purified compound were obtained by analysis with a Bruker Avance III-500 spectrometer.

### Determination of the MICs of HA against different bacteria.

Stock solutions (128 μg/mL) of HA and ampicillin (Qisong Biotech, Shanghai, China; dissolved in sterile water) were prepared and diluted in 2-fold series in liquid LB medium (for S. aureus, E. faecalis, *C. nuruki*, and E. coli) and MRS (for *L. plantarum*, *A. persici*, and *L. mesenteroides*). The drug-containing aliquots (100 μL each) were loaded into the 96-well plates with three replicates for each compound concentration. The freshly prepared bacterial cells were adjusted with either LB or MRS broth to the concentration of ca. 2 × 10^5^ CFU/mL and loaded (100 μL each) into the drug-containing cells. The LB medium samples were incubated at 37°C and the MRS medium samples at 30°C in a rotary shaker at 180 rpm for 20 h to determine the MICs of HA and ampicillin against each bacterium. The wells without antibiotics were included as controls. To further determine the bacteriostatic or bactericidal effect of HA, we grew *L. plantarum*, S. aureus, and *A. persici* at 0× (control), 1×, 2×, and 4× MIC of HA for 20 h, and the suspensions (50 μL each) were then plated on LB plates for 20 h to determine the formation of bacterial colonies. There were three replicates for each treatment.

### Cross inhibition assays between fungi and bacteria.

The WT and mutant spores of *M. robertsii* were collected in 0.05% of Tween 20 from the 2-week-old PDA plates. The WT spore suspensions (each at a final concentration of 5 × 10^6^ conidia/mL) were added with S. aureus cells at final OD_600_ values of 0.05 and 0.2 to LB medium. The samples were incubated at 25°C and 210 rpm for 24 h prior to being photographed to show fungal inhibition of bacterial growth. The WT spores were also heat killed by boiling for 5 min and used for inhibition assays. S. aureus cultures without fungal spores were included as controls. The mixtures (100 μL each) of the fungal spores and bacterial cells (all at a final OD_600_ of 0.2) were also inoculated on LB agar for 24 h. There were three replicates for each sample.

For assaying the bacterial inhibition of fungal spore germinations, different amounts of bacterial cell were first assayed to determine the proper starting density for each species. The number of cells of each G+ bacterium that could result in a difference in spore germination between the WT and the Δ*MrHelA* mutant was used in the assays. For G− bacteria, the numbers of cells that could inhibit an average of >30% spore germination were used in the assays. After these trial assays, the bacterial cells were adjusted in LB broth (4 mL each in test tubes) at final OD_600_ values of 0.01 for S. aureus, 0.005 for E. faecalis and *C. nuruki*, 0.05 for *L. plantarum*, *L. mesenteroides*, and *A. persici*, and 0.0001 for E. coli. Fungal spores were then added each at a final concentration of 5 × 10^6^ conidia/mL. The samples were incubated at 25°C and 210 rpm for 12 h to determine the spore germination rate of each strain. Spore germinations in LB broth without bacteria were included as mock-treated controls. There were three replicates for each sample, and 35 microscopic fields were recorded for each replicate. Two-tailed Student’s *t* tests were conducted to determine the difference in germination between the WT and mutant strains and between the mock-treated control and bacterial treatments.

To determine whether the presence of bacterial cells could affect fungal penetration, we mixed the spore suspensions (each at 1 × 10^6^ conidia/mL) with the cells of different G+ and G− bacteria (each at a final OD_600_ of 0.02) for inoculation (2 μL each) of cellophane membranes laid on minimum medium (45). Four days postinoculation, the membranes were carefully removed, and the samples were incubated for six additional days to assess the outgrowth of fungal cultures. There were five repeats for each treatment.

### Insect bioassays.

To exclude the sex dimorphisms in immune responses (53), we used 3-DPE D. melanogaster (isogenic W1118 line) and *D. suzukii* (collected from a waxberry field and maintained in the lab) male adults for survival assays using the WT and mutant spores of *M. robertsii*. In addition, last-instar larvae of the wax moth (G. mellonella; Keyun, China) were challenged for both topical infection and injection assays. The flies were anesthetized with CO_2_ and then left on ice prior to immersion assays. The spore suspensions of 2 × 10^7^ conidia/mL were prepared in 0.05% Tween 20 for topical infections of insects by immersion in suspensions for 30 s. Conventionally reared 3- and 10-DPE male fruit flies, 3-DPE *D. suzukii* males, and wax moth larvae were used for natural infections. The 3-DPE males of both *Drosophila* flies were also used for injections (10 nl of a suspension containing 1 × 10^7^ conidia/mL) using a microinjector (Nanoject III; Drummond, Broomall, PA). The wax moth larvae were injected (5 μL a suspension containing 5 × 10^4^ conidia/mL) in the second proleg using a hand microapplicator (Burkard, Hertfordshire, UK).

For gnotobiotic bioassays, axenic fruit flies were prepared as described before and checked by PCR analysis using the primers 27F and 1492R (29, 54). 3-DPE sterile males were used for topical infection with the concentrations of the WT and mutant spore suspensions indicated above. In addition, the spore suspensions were added with the final amounts of the *C. nuruki* (OD_600_ values of 0.2 and 0.5) and *L. plantarum* (OD_600_ values of 0.05, 0.1, and 0.5) cells for immersion of sterile males. There were more than 70 flies and 45 caterpillars used for each treatment. Insect survival was recorded every 12 h, and the differences in survival between strains and between treatments were determined by Kaplan-Meier analysis and log-rank tests (55).

### SEM analysis.

10-DPE flies of D. melanogaster were immersed in the WT strain spore suspension and 0.05% Tween 20 for 30 s. After treatment for 18 h, the flies were freeze killed for SEM observations using a field emission scanning electron microscope (Merlin Compact VP; Zeiss) as described before (29).

### Fly surface bacterial load analysis.

The CFU formation and quantitative microbiota analysis was performed as we described before (29). Briefly, conventionally reared 3- and 10-DPE male fruit flies were immersed in the spore suspensions (2 × 10^7^ conidia/mL) of the WT and mutant strains for 30 s. Control flies were treated with 0.05% Tween 20. After treatments for 16 h, the flies were collected, anesthetized with CO_2_, and then placed on ice. Groups of 10 flies were washed in 1 mL of phosphate-buffered saline (pH 7.4) by vortexing for 1 min, and the wash solutions were diluted 10 times. Aliquots (100 μL each) were inoculated on LB agar plates (9 cm in diameter) for 2 days at 28°C. The CFU were counted and converted to units per fly. There were 16 replicates for each sample, and the difference between treatments was determined by one-way ANOVA. The washed bacterial samples from the 3-DPE males were also used for quantitative microbiome analysis by adding the pUC57 plasmid (0.05 pg each) containing the synthetic stuffer sequence as a spike-in standard (56). The PCR products were amplified with the universal primers 515F and 806R for the generation of amplicon libraries and sequencing analysis by Biozeron (Shanghai, China). Data analysis was performed as we described before (29).

### Data availability.

Bacterial 16S rRNA sequencing fastq data have been deposited in the SRA (Sequencing Read Archive) database with the BioProject accession number PRJNA836348 (SAMN28157742 to SAMN28157765).
