# Supplementary material for: Production of Helvolic Acid in Metarhizium Contributes to Fungal Infection of Insects by Bacteriostatic Inhibition of the Host Cuticular Microbiomes
Source: Microbiol Spectr. 2022 Sep 1;10(5):e02620-22. doi: 10.1128/spectrum.02620-22 (PMC9602595; doi:10.1128/spectrum.02620-22)
Supplement: Supplemental file 1 — Supplemental material. Download spectrum.02620-22-s0001.pdf, PDF file, 1.4 MB [file spectrum.02620-22-s0001.pdf]

Supplementary materials

Supporting figures:

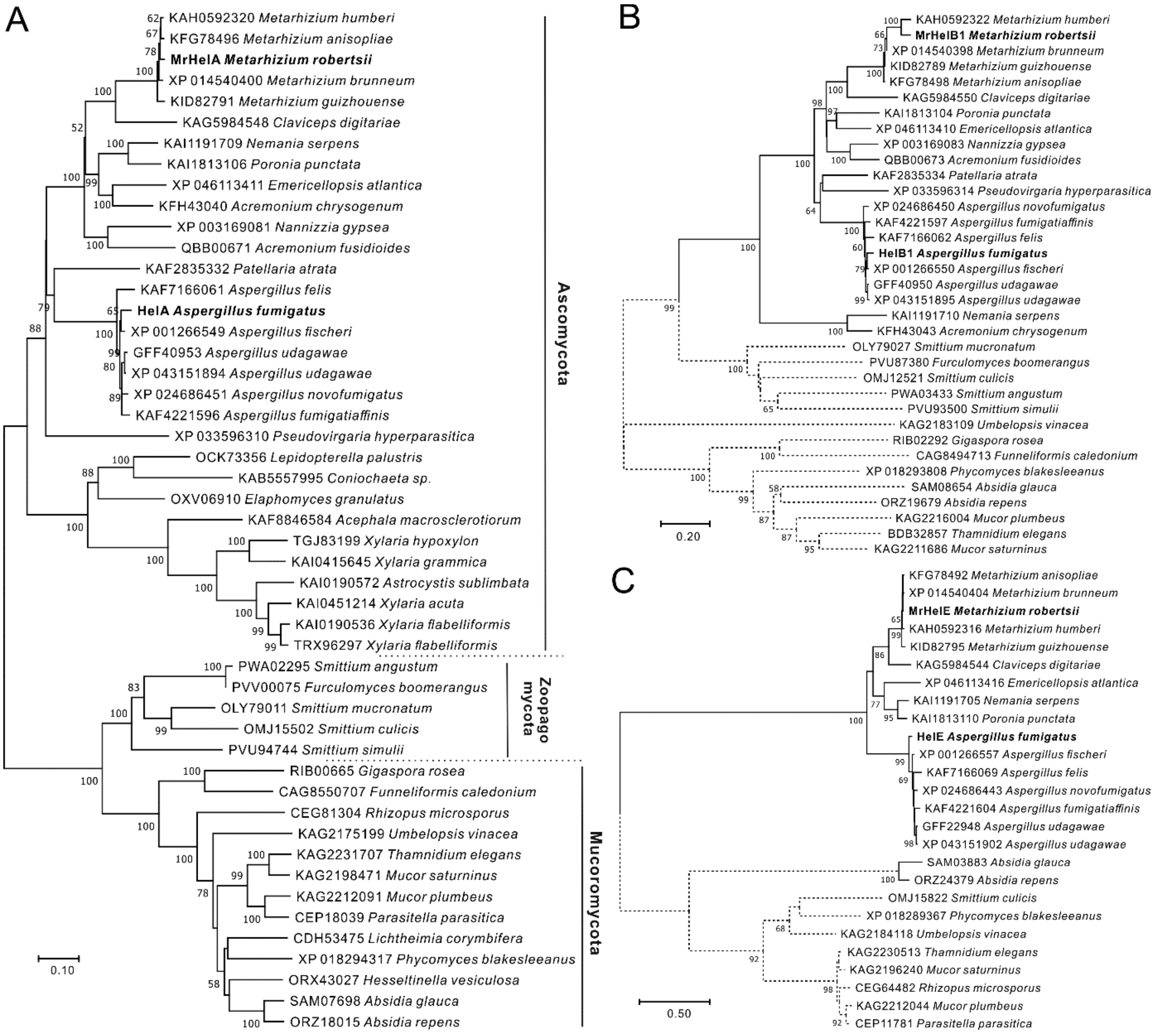

FIG S1 Phylogeny analysis of the putative HA-biosynthesis-related terpene cyclase (A), cytochrome P450 (B) and ketosteroid-dehydrogenase (C) enzymes encoded by different fungi. The neighbor-joining trees were generated using the Dayhoff model for amino acid substitution and 500 bootstrap replicates. The dash-lines mean the homologous genes of different fungi that do not cluster with the core terpene cyclase gene.

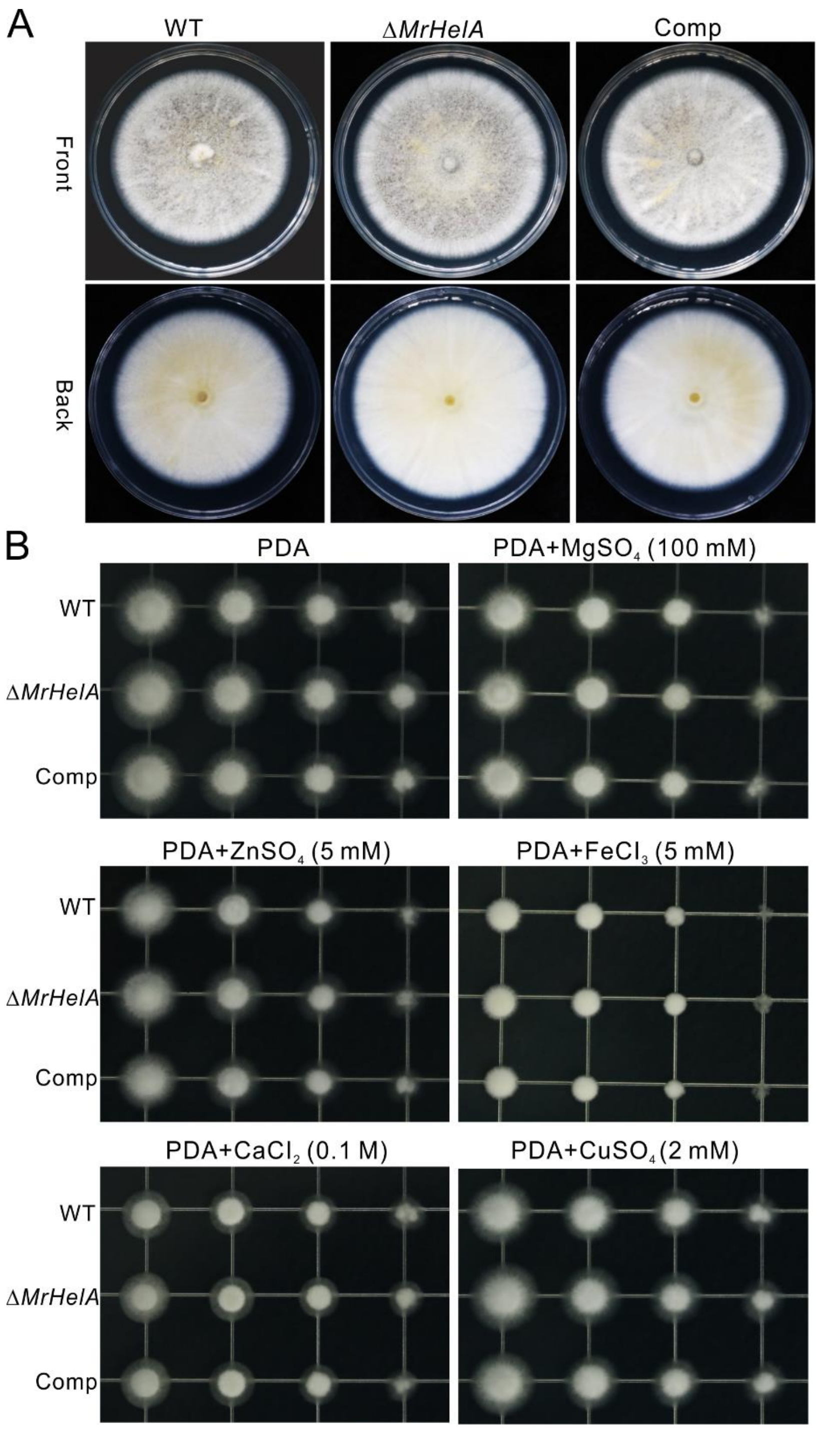

FIG S2 Growth and salt stress challenges of the WT and mutant strains. (A) Growth phenotyping of the WT and mutant strains inoculated on PDA for two weeks. (B) No obvious difference in resistance against the salt stresses between the WT and mutant strains. Fungal spore suspensions were diluted in 10-fold series (from 1×10^7 to 1×10^4 conidia/ml) and inoculated (2 μl each) on media for three days.

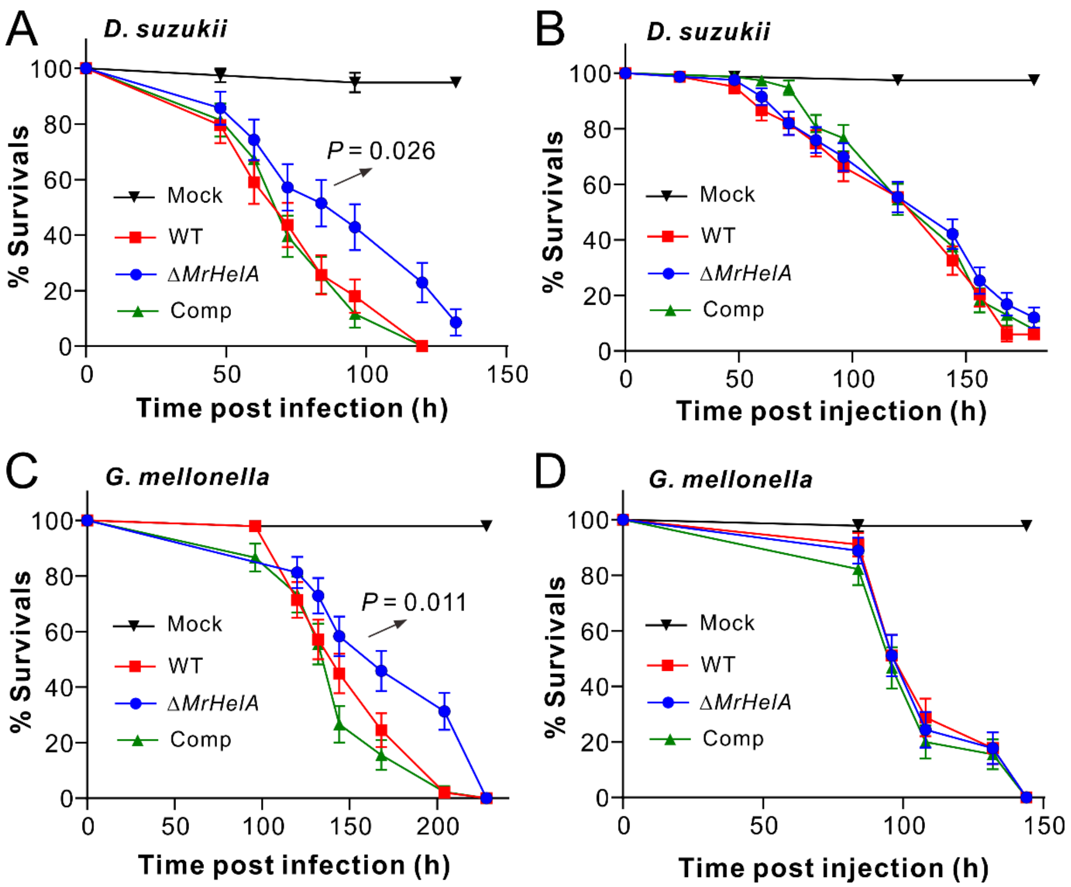

FIG S3 Insect survival assays. (A, B) Survival of *D. suzukii* males after topical infection (A) and injection (B) with different *M. robertsii* strains. (C, D) Survival of the wax moth (*G. mellonella*) larvae after topical infection (C) and injection (D) with different *M. robertsii* strains. Plotted values are mean ± SEM. Mock control insects were treated with 0.05% of Tween 20. The differences between treatments were determined by log-rank test. The arrowed *P* values shown in panels mean the difference level between the WT and Δ*MrHelA* treatments.

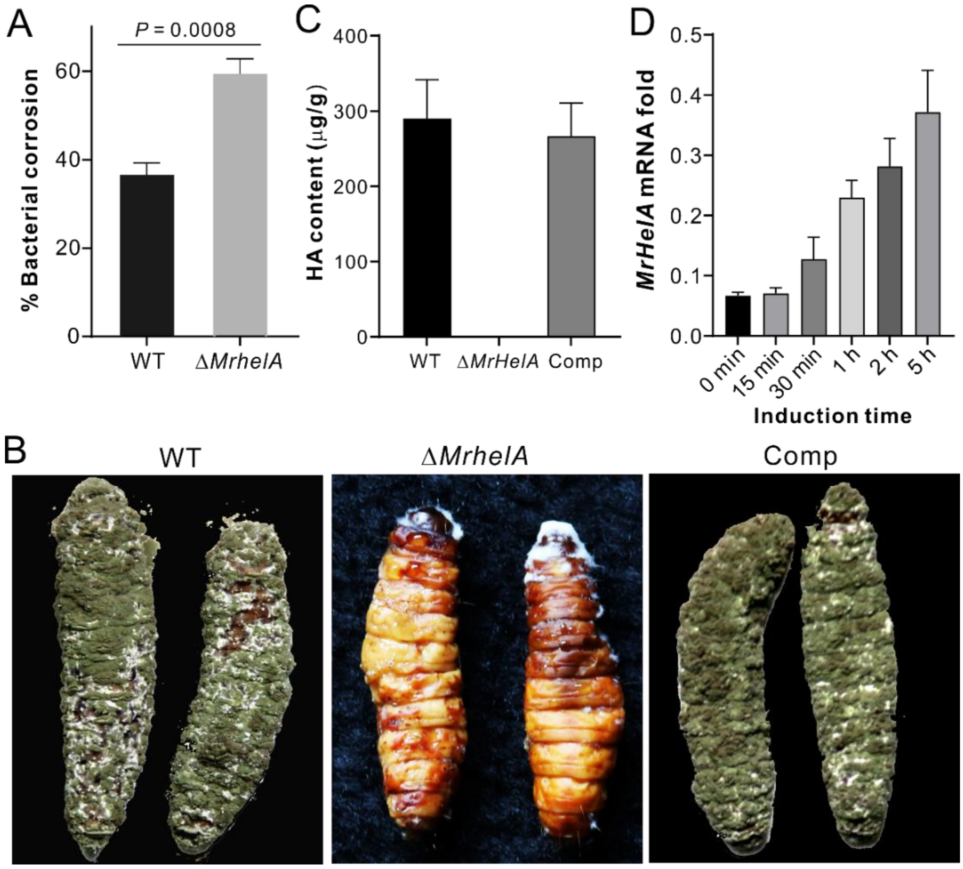

FIG S4 Characterization of different features. (A) Percentage of the wax moth cadavers corroded by bacteria after topical infections by the WT and Δ*MrHelA* strains. The difference between strains was determined by two-tailed Student's *t*-test. (B) Phenotypes of the cadavers of the wax moth larvae after killed by the WT and mutant strains. In contrast to the mycoses of cadavers by the WT and complementation (Comp) strains, the insects killed by Δ*MrHelA* were largely colonized by bacteria without fungal sporulation. (C) Quantification of HA production in insect cadavers. The mycosed cadavers were freeze dried, weighted and extracted for HPLC analysis. (D) Induction of *MrHelA* expression by *S. aureus*. Fungal spores were inoculated in LB broth for 24 hours, and the cells of *S. aureus* were then added into the aliquoted samples at a final OD_{600} value of 0.4 for different times as indicated.

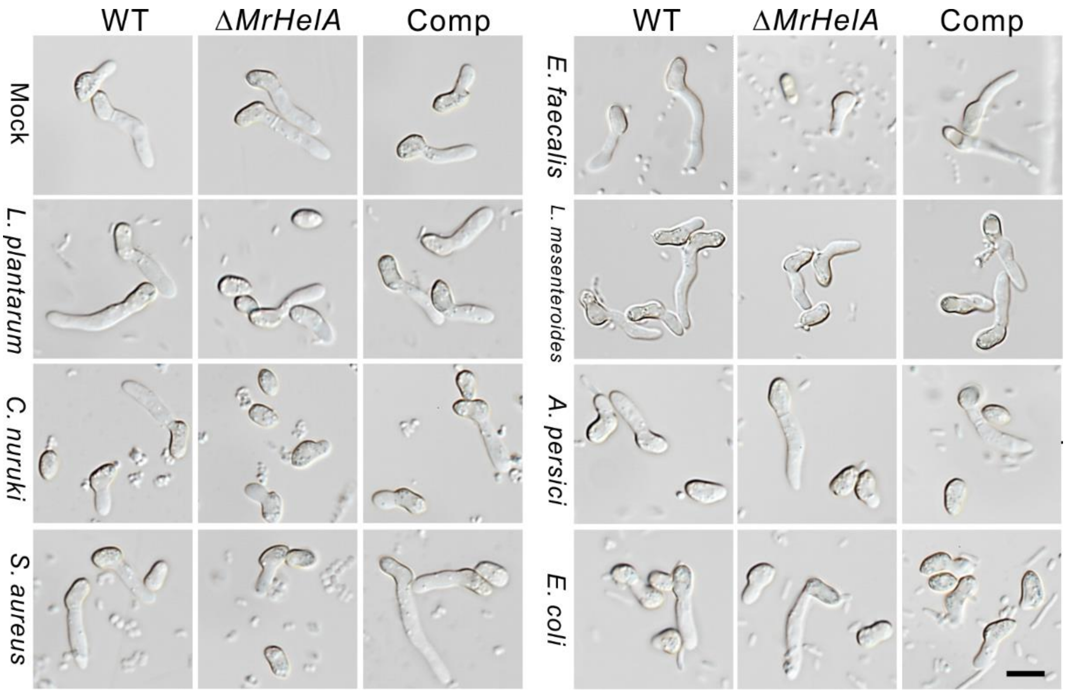

FIG S5 Microscopic imaging of fungal spore germinations in the presence or absence of bacterial cells. The representative photos were taken 12 hours post co-inoculation in LB broth. Bar, 5 μm.

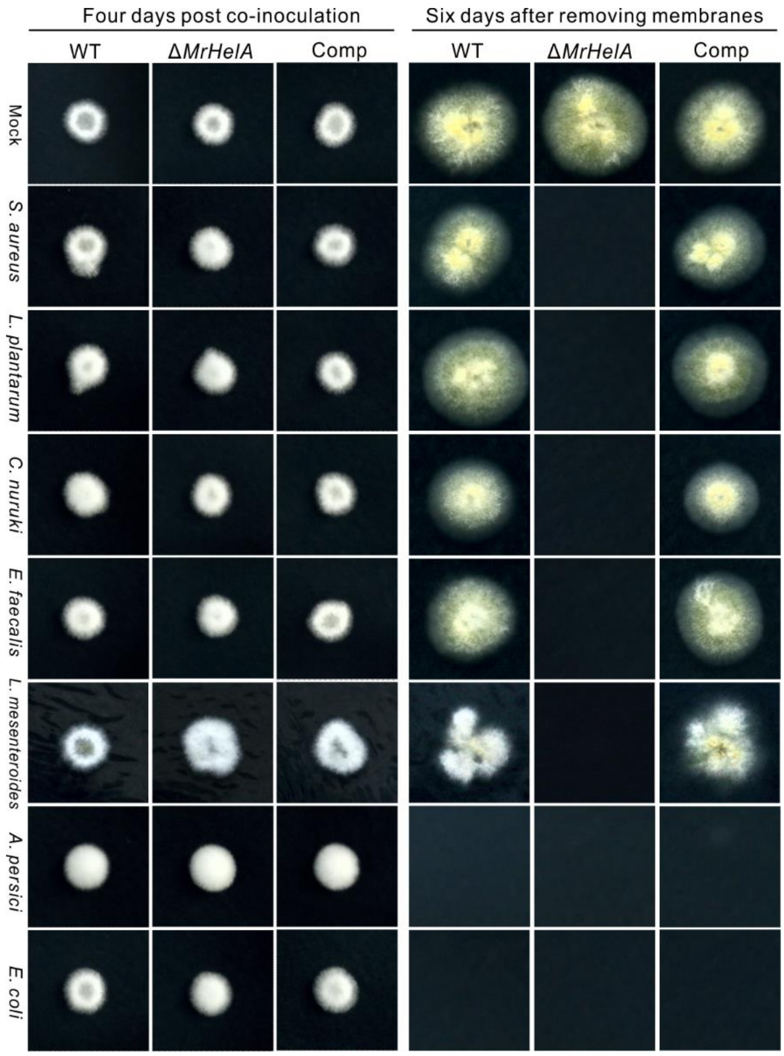

FIG S6 Bacterial antagonism against fungal penetration of cellophane membranes. The WT and mutant spore suspensions (each at 1×10^6 conidia/ml) were added with the cells of different bacteria (each at a final OD_{600} of 0.02) prior to being inoculated on the cellophane membranes (2 μl each) for four days. The membranes were then carefully removed and the samples were kept for incubation for six additional days. Figures showing the representative of five repeats for each sample.

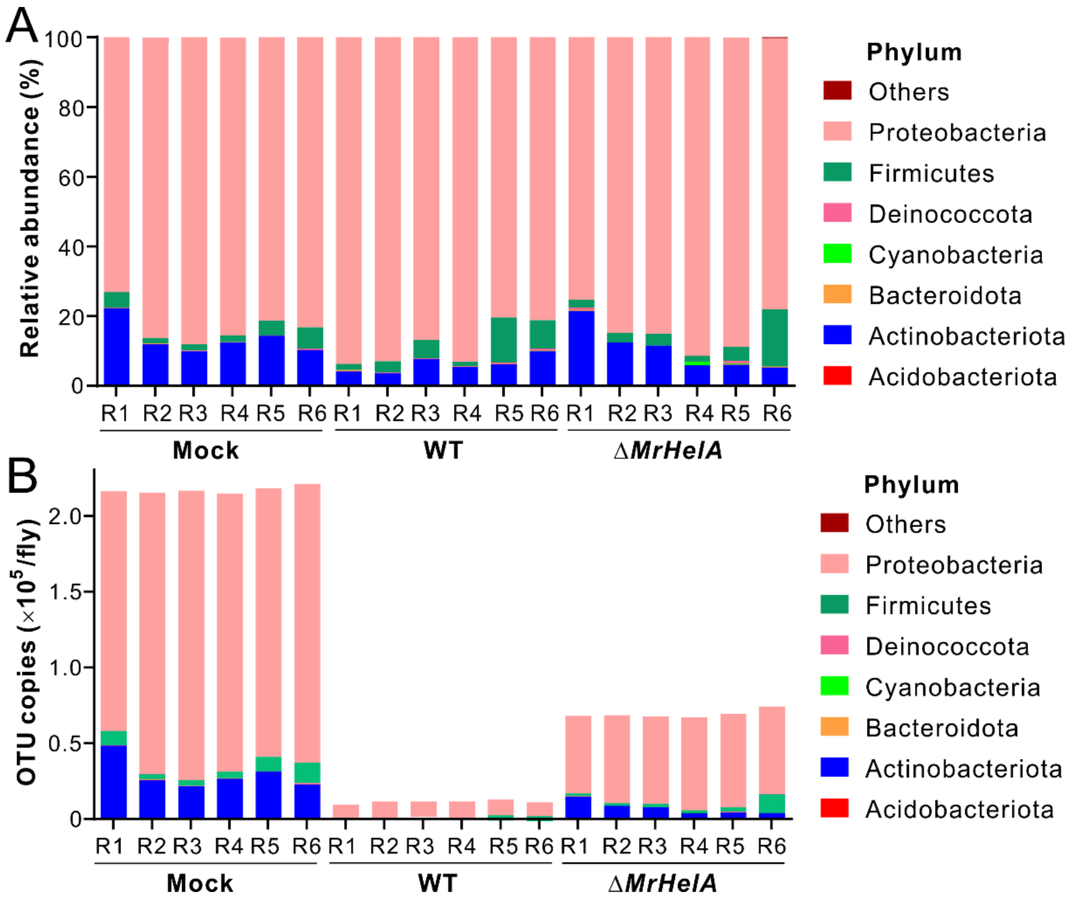

FIG S7 Quantitative analysis of fly surface microbiomes showing the relative (B) and quantified (C) abundance variations between different treatments at the bacterial phylum level. The flies were immersed in 0.05% Tween 20 (Mock) and spore suspensions of the WT and Δ*MrHelA* for 30 seconds. After treatments for 16 hours, the flies were collected (10 insects per replicate) for washing of surface bacteria for plating, and the aliquots were used for 16S *rRNA* amplification and library sequencing.

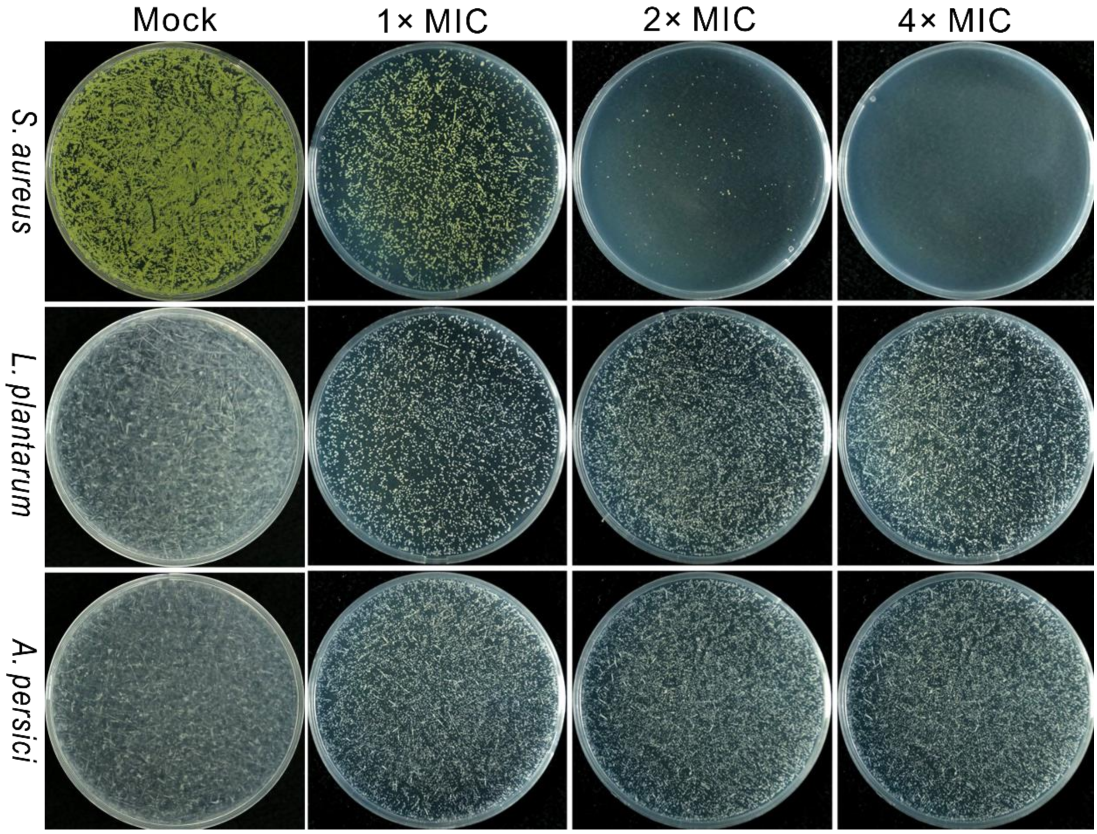

FIG S8 Examination of the bacteriostatic or bactericidal effect of HA against different bacteria. The G+ bacteria *S. aureus* and *L. plantarum,* and G- bacterium *A. persici* were incubated in the medium containing the respective 0× (mock control), 1×, 2× and 4× MIC doses of HA for 20 hours, and each sample (50 μl) was then plated on the LB agar plates for 20 hours.

TABLE S1 Conservation of the helvolic acid biosynthetic gene clusters encoded by *A. fumigatus* and *Metarhizium* species.

A. fumigatus	***M. robertsii***	**Putative function**	**Identity* (%)**	***M. anisopliae***	***M. brunneum***	***M. guizhouense***	***M. humberi***
HelA	EXV00365, *MrHelA*	Oxidosqualene cyclase	64.4	KFG78496	XP_014540400	KID82791	KAH0592320
HelB1	EXV00363, *MrHelB1*	Cytochrome P450	64.7	KFG78498	XP_014540398	KID82789	KAH0592322
HelB2	EXV00367, *MrHelB2*	Cytochrome P450	58.4	KFG78494	XP_014540402	KID82793	KAH0592318
HelC	EXV00366, *MrHelC*	Short-chain dehydrogenase	62.0	KFG78495	XP_014540401	KID82792	KAH0592319
HelB3	EXV00371, *MrHelB3*	Cytochrome P450	63.3	KFG78490	XP_014540406	KID82797	KAH0592314
HelD1	EXV00370, *MrHelD1*	Acyltransferase	47.5	KFG78491	XP_014540405	KID82796	KAH0592315
HelB4	EXV00364, *MrHelB4*	Cytochrome P450	64.6	KFG78497	XP_014540399	KID82790	KAH0592321
HelD2	EXV00368, *MrHelD2*	Acyltransferase	42.0	KFG78493	XP_014540403	KID82794	KAH0592317
HelE	EXV00369, *MrHelE*	3-Ketosteroid-Δ^1-dehydrogenase	59.3	KFG78492	XP_014540404	KID82795	KAH0592316

*, identity at the amino acid level between the relative proteins encoded by *A. fumigatus* and *M. robertsii*.

TALBE S2 NMR spectroscopic data of helvolic acid in pyridine-d5*.

No.	δH (mult., J in Hz)	δC
1	7.31, d (10.2)	158.0
2	6.01, d (10.0)	127.7
3		201.1
4	2.91, dp	40.6
4-CH3	1.35, d (6.6)	12.9
5	2.54, d	47.2
6	5.56, s	74.3
6-OCOCH3		169.4
6-OCOCH3	2.12, s	20.6
7		209.7
8		53.0
8-CH3	1.15, s	18.3
9	2.68	42.0
10		38.5
11	1.55	23.9
	1.87	
12	1.85	26.3
	2.47	
13	2.68, d	49.1
14		47.0
15	2.20, d (14.4)	41.4
	2.42	
16	6.44, d (8.3)	74.1
16-OCOCH3		170.4
16-OCOCH3	1.91, s	20.5
17		144.6
18	1.12, s	18.1
19	1.45, s	27.5
20		133.0
21		172.9
22	2.83	29.4
	2.93	
23	2.42	29.1
	2.57	
24	5.38, t	124.3
25		132.3
26	1.68, s	17.8
27	1.17, s	25.8

*, The data were recorded at 400 and 100 MHz for ^{1}H and ^{13}C, respectively.

TABLE S3 The PCR primers used in this study.

**Gene**	**Primers**	**Primer sequences**	**RE***	**Note**
MrHelA	UF	GCTTGATATCGAATTCCAGAACTTGTCCGTGGTG	PstI	Gene deletion
	UR	CATCTTCTGTCGACGTCAAGGGATGTCTGTTCCTCT		
	DF	TCACCGAGATCTGACATGTGGATGTGCAGAGAG	BcuI	
	DR	CGCGGTGGCGGCCGCTCTAGAGGACCGTTGATTCTACTG		
	OutF	TTCGTTGCAAAGGCGTATCC	/	Deletion mutant verification
	OutR	CATGAACCCTTGGTCGGCT		
	InF	TGGGGCAAGTTCTGGATG	/	
	InR	CTTCGACCTGGAACAAGG		
	CompF	TCACGTCGACTAGTTGGATGCAGTCATTAGATCGT	BcuI	Gene complementation
	CompR	CCGCTCTAGAACTAGCTACTGTTTCTGCAATTCGC		
	qtF	CTGCTTCACCTATGCCACCA	/	qRT-PCR
	qtR	GTCTTGGACGTACCGCTTGA		
β-tubulin	TF	GTCACCACATGCTTGCGTTT	/	qRT-PCR reference
	TR	GTCGAACATCTGCTGGGTGA		
Bacterial 16S *rRNA* genes	27F	AGAGTTTGATCCTGGCTCAG	/	Axenic fly check
	1492R	TACGGYTACCTTGTTACGACTT		
	515F	GTGCCAGCMGCCGCGG	/	Microbiome sequencing
	806R	GGACTACNNGGGTATCTAAT		

*RE, restriction enzyme.
